# Hindbrain insulin controls feeding behavior

**DOI:** 10.1016/j.molmet.2022.101614

**Published:** 2022-10-13

**Authors:** Kim Eerola, Francesco Longo, Thomas M. Reinbothe, Jennifer E. Richard, Olesya T. Shevchouk, Lorena López-Ferreras, Devesh Mishra, Mohammed Asker, Johan Tolö, Caroline Miranda, Saliha Musovic, Charlotta S. Olofsson, Patrik Rorsman, Karolina P. Skibicka

**Affiliations:** 1 Institute for Neuroscience and Physiology, University of Gothenburg, Sweden; 2 Wallenberg Centre for Molecular and Translational Medicine, University of Gothenburg, Sweden; 3 Department of Nutritional Sciences and The Huck Institutes of the Life Sciences, Pennsylvania State University, University Park, PA, USA; 4 Unit of Integrative Physiology and Pharmacology, Institute of Biomedicine, University of Turku, Finland; 5 Oxford Centre for Diabetes, Endocrinology and Metabolism, University of Oxford, Churchill Hospital, Oxford, UK

**Keywords:** Hindbrain, Dorsal vagal complex, Food intake, Diet-induced obesity, Insulin

## Abstract

**Objective:**

Pancreatic insulin was discovered a century ago, and this discovery led to the first lifesaving treatment for diabetes. While still controversial, nearly one hundred published reports suggest that insulin is also produced in the brain, with most focusing on hypothalamic or cortical insulin-producing cells. However, specific function for insulin produced within the brain remains poorly understood. Here we identify insulin expression in the hindbrain's dorsal vagal complex (DVC), and determine the role of this source of insulin in feeding and metabolism, as well as its response to diet-induced obesity in mice.

**Methods:**

To determine the contribution of Ins2-producing neurons to feeding behavior in mice, we used the cross of transgenic RipHER-cre mouse and channelrhodopsin-2 expressing animals, which allowed us to optogenetically stimulate neurons expressing Ins2 *in vivo*. To confirm the presence of insulin expression in Rip-labeled DVC cells, in situ hybridization was used. To ascertain the specific role of insulin in effects discovered via optogenetic stimulation a selective, CNS applied, insulin receptor antagonist was used. To understand the physiological contribution of insulin made in the hindbrain a virogenetic knockdown strategy was used.

**Results:**

Insulin gene expression and presence of insulin-promoter driven fluorescence in rat insulin promoter (Rip)-transgenic mice were detected in the hypothalamus, but also in the DVC. Insulin mRNA was present in nearly all fluorescently labeled cells in DVC. Diet-induced obesity in mice altered brain insulin gene expression, in a neuroanatomically divergent manner; while in the hypothalamus the expected obesity-induced reduction was found, in the DVC diet-induced obesity resulted in increased expression of the insulin gene. This led us to hypothesize a potentially divergent energy balance role of insulin in these two brain areas. To determine the acute impact of activating insulin-producing neurons in the DVC, optic stimulation of light-sensitive channelrhodopsin 2 in Rip-transgenic mice was utilized. Optogenetic photoactivation induced hyperphagia after acute activation of the DVC insulin neurons. This hyperphagia was blocked by central application of the insulin receptor antagonist S961, suggesting the feeding response was driven by insulin. To determine whether DVC insulin has a necessary contribution to feeding and metabolism, virogenetic insulin gene knockdown (KD) strategy, which allows for site-specific reduction of insulin gene expression in adult mice, was used. While chow-fed mice failed to reveal any changes of feeding or thermogenesis in response to the KD, mice challenged with a high-fat diet consumed less food. No changes in body weight were identified, possibly resulting from compensatory reduction in thermogenesis.

**Conclusions:**

Together, our data suggest an important role for hindbrain insulin and insulin-producing cells in energy homeostasis.

## Highlights

• The hindbrain's DVC is a potential source of insulin-producing cells.
• Optogenetic activation of hindbrain insulin-producing cells increases food intake.
• This hyperphagia is dependent on brain insulin receptors.
• Diet-induced obesity alters the function and increases the levels of hindbrain insulin.

## 1 Introduction

Considering the staggering, unabated, and costly (140 billion per year [1]) rates of obesity in the Western world, there is tremendous interest in understanding the neural circuits controlling feeding, and identifying pharmacological targets to reduce overeating. A wealth of data indicates that insulin is produced in the brain [[2], [3], [4], [5], [6], [7], [8], [9], [10], [11], [12], [13], [14], [15], [16], [17], [18]]; nevertheless, the idea of brain-produced insulin is still controversial. Pancreatic insulin reaches the central nervous system (CNS) via a saturable transport across the blood–brain barrier (BBB), but with restricted access [19,20] that prioritizes circumventricular organs lacking a BBB. Although insulin is unequivocally found in brain tissue, the exact concentrations of insulin are a matter of debate with some laboratories finding 10–100 times higher concentrations in the brain parenchyma compared to plasma levels, depending on the area of the brain [15]. While others report concentrations similar to those found in the plasma but still widely varying in different CNS regions [15,21,22] supporting the idea of locally produced insulin.

Compared to current knowledge on the role of insulin in peripheral tissues, like muscle or adipose tissue, much less is known about the impact of insulin on the CNS. Insulin acts on the brain to alter energy balance. Its infusion into the brain ventricles reduces food intake and body weight in primates and rodents, but promotes lipogenesis and peripheral fat accumulation [23,24]. Furthermore, insulin decreases sucrose intake as well as sucrose reward in rats [25,26]. Critical role of brain insulin receptors (InsR) is indicated by the hyperphagia, obesity, and increased fat mass resulting from knockout of CNS InsR [[27], [28], [29]]. CNS InsR offer a complementary mechanism to affect glucose homeostasis. Central delivery of insulin, unlike pancreatic insulin directly reaching peripheral organs, does not affect glucose uptake in skeletal muscle or adipose tissue. Yet, brain administration of insulin reduces blood glucose levels, and this effect is abolished by removal of the liver [30,31]. Hypothalamic InsR activation regulates hepatic glucose production, likely via vagal efferents [32]. Specifically this small InsR population in the hypothalamus is critical to normal glucoregulation since physiologically elevated insulin fails to suppress hepatic glucose production when hypothalamic InsR are silenced [28,32]. Knockdown of InsR in the ventromedial hypothalamus triggers hepatic insulin resistance and glucose intolerance [33]. Notably, the manipulations of brain InsR described above are not designed to discriminate between central vs. peripheral source of insulin. Based on these broad and potent effects of InsR manipulation in the brain, there is an intriguing possibility that manipulations of brain-produced insulin will affect appetite, body fat and glucoregulatory homeostasis, a hypothesis pursued here.

Obesity affects numerous steps governing insulin action, including production in the pancreas, transport, and transmission of the insulin message by target cells [34,35]. Increased pancreatic insulin production correlates with type 2 diabetes and obesity [35]. Yet to date the literature offers little insight into brain insulin expression in diet-induced obesity. Thus, here we will determine the impact of diet-induced-obesity (DIO) on brain insulin gene expression in brain areas well-established to have a role in energy balance regulation, the hypothalamus and the dorsal vagal complex (DVC).

The role of insulin-gene (ins2) positive neurons (but not insulin produced by these cells) was previously investigated, by using the rat insulin promoter (Rip), active in the CNS, and therefore used as a means to select the cellular target of interest. The first study suggested that these hypothalamic neurons are catabolic in nature [36]; the second implied that they are anabolic [37]. Another study reported a differential label of cells in the brain in different insulin gene based manipulations [2]. A limiting factor of these studies is their indiscriminate impact on all insulinergic neurons/CNS-Rip-cells, potentially leading to conflicting data. Moreover, some studies reported that cre recombinase insertion alone leads to glucoregulatory disturbances [38] (for Rip_MAG_-cre [39]). Importantly, this has not been detected in other Rip (in Rip_HER_-cre [40]) or Ins2-cre lines [2]. Therefore, here we use Rip_HER_-cre [40] mice, shown to express cre in the pancreas and in discrete CNS nuclei [37], but not displaying cre-insertion induced glucoregulatory disturbances [40].

To the best of our knowledge, the physiological role of CNS-produced insulin has not been directly investigated and insulin promoter-driven manipulations deliver a lack of consensus. To determine the contribution of Ins2-producing neurons to feeding behavior in mice, we used the cross of transgenic Rip_HER_-cre mouse and channelrhodopsin-2 expressing animals, which allows us to optogenetically stimulate neurons expressing Ins2 *in vivo*. To ascertain the specific role of insulin in effects discovered via optogenetic stimulation a selective, CNS applied, insulin receptor antagonist was used. To understand the physiological contribution of insulin made in the hindbrain a virogenetic knockdown strategy was used. Our overarching aim was to identify novel insulin-expressing cell populations outside of the hypothalamus, and determine their physiological role in health and during diet-induced obesity challenge in mice.

## 2 Materials and methods

**Animals:** Mice expressing Channelrhodopsin-2(H134R) and YFP under the rat insulin promoter (Rip-TG), previously described in [41] were used to assess central RIP expression. The mice were bred by crossing heterozygous B6; 129S Channelrhodopsin-2(H134R)-YFP mice (Jackson laboratories Ai32 #012569) [42] and hemizygous Rip-Cre mice [43] to obtain Rip-ChR2-YFP. For optogenetic experiments, heterozygous male and female Ai27D B6; 129S Chr2(H134R)-TdTomato mice were crossed with Rip-Cre to obtain Rip2-ChR2-TdTomato mice. C57BL/6J were used to assess Ins2 gene expression and for the assessment of insulin gene KD (Ins2-KD) experiment. All mice were housed under a 12h light/dark cycle, in individual cages after surgery with ad libitum access to chow and water, unless otherwise stated. All studies were carried out with ethical guidelines and permissions from the Animal Welfare Committee of the University of Gothenburg, in accordance with legal requirements of the European Community (Decree 137/15). All efforts were made to minimize suffering.

**Gene expression:** Five-week-old male C57BL/6J mice were fed standard rodent chow (Global Diet #2016; Harlan-Teklad) or high-fat diet (HFD; catalog #D12492; Research Diets Inc.; 60% kcal from fat) for 8 weeks before isolation of brain regions (Figure 1) by using a 1mm/section brain matrix for mice (cat. RBM-2000C, ASI instruments, Inc. Warren, MI, USA with a mouse brain atlas as reference [44]. Total RNA was extracted using RNeasy Lipid Tissue Mini kit (QIAGEN) by removing genomic DNA by deoxy ribonuclease treatment (QIAGEN). A Nanodrop 1000 (NanoDrop Technologies) was used to spectrophotometrically assess the concentration and quality of the isolated RNA was synthesized using iScript cDNA Synthesis kit (Bio-Rad). Real-time RT-PCR was performed using TaqMan Assay including selective primer sets for the Ins2-gene (Mm00731595_gH Ins2) and beta-actin (Mm00607939_s1 Bact). The ΔΔCt method [45] was used to compare the relative gene expression between treatment groups (n = 8–10) using beta-actin as a reference. As described by Livak et al. [45] in more detail, in the ΔΔCt method the delta Ct is first calculated and then the resulting value of controls (here chow-fed mice) is normalized to 1.

**Figure 1:**
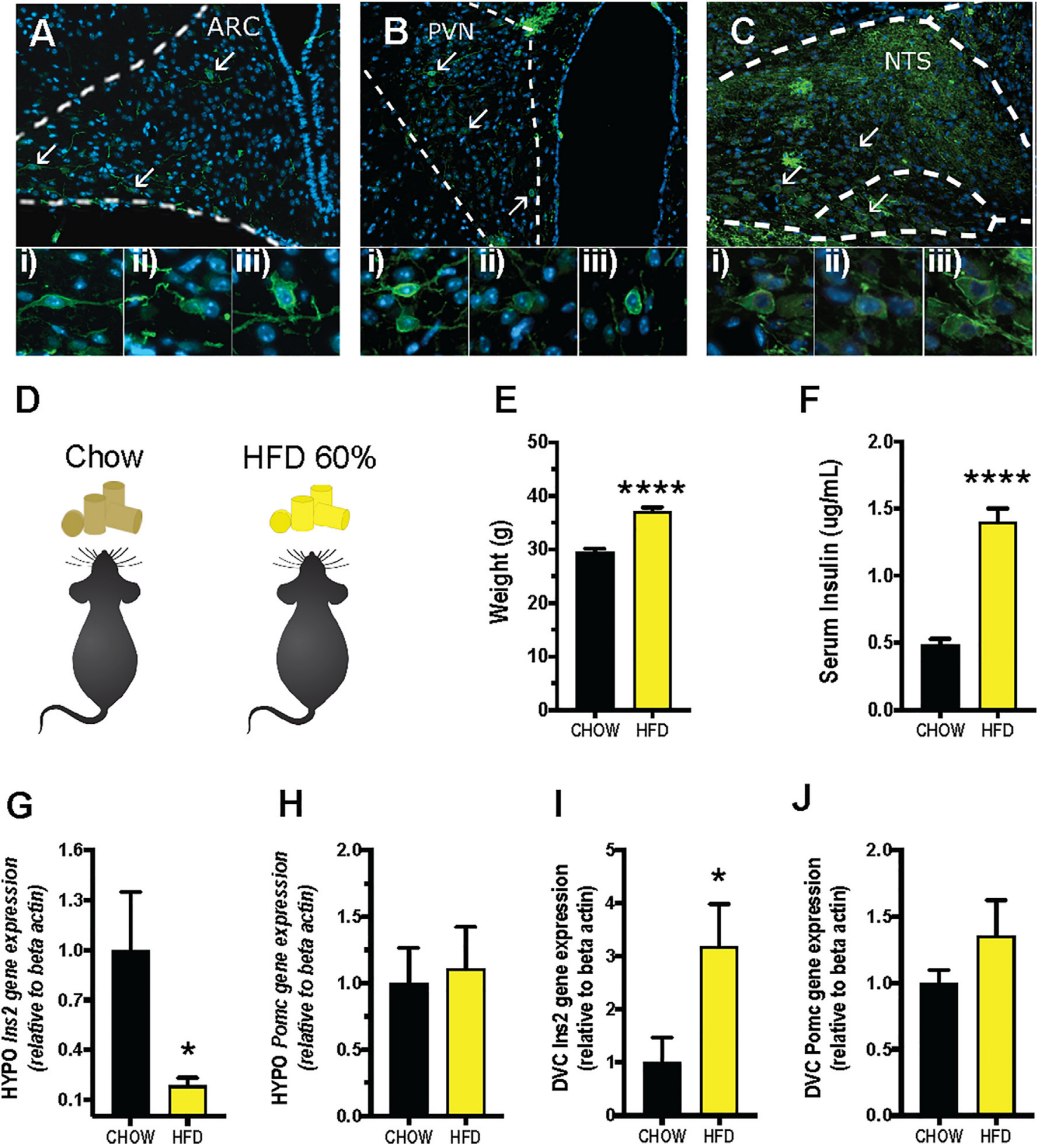
**Rat Ins2 promoter cells, found in the hypothalamus and hindbrain, are affected by diet-induced obesity in a neuroanatomically divergent manner**. 20x epifluorescent images of A: YFP- expression in the arcuate nucleus (ARC), B: paraventricular nucleus (PVN) and C: the nucleus of the solitary tract (NTS) in Rip-Chr2-YFP mice. Lower magnification image and corresponding mouse brain atlas section of NTS are shown in Figs. S1 and S2. Higher magnification images showcasing individual cells from each area are also shown in Fig. S3. D: Eight weeks of high-fat diet maintenance induced E: weight gain measured at 8wks of diet exposure and F: increased serum insulin levels. G: Expression of *Ins2*-and H: *Pomc*-gene expression in the hypothalamus, and I: *Ins2*-and J: *Pomc-*gene expression in the brainstem of mice on the HFD for eight weeks. n = 8–10, ∗p < 0.05, ∗∗∗∗p < 0.00001 students t-test. YFP expression is visualized in green, and DAPI (nuclear stain) in blue.

**In situ hybridization/RNAscope:** Expression of Insulin 2 mRNA in YFP-expressing cells was detected by using RNAscope™ multiplex fluorescent kit (Advanced Cell Diagnostics, Biotechne®, Hayward, CA) on Rip-ChR2-YFP mouse brain sections and performed as previously described [46,47]. Briefly, the fresh frozen mouse brain was embedded in a cryo-embedding medium (OCT) and then coronally sectioned at 16 μm. Mounted sections were then fixed in paraformaldehyde (PFA) 4%, dehydrated in graded ethanol (50%, 70%, and 100%), digested with protease, and followed by hybridizations with target-specific probes. Mm-Ins2-O1-C2 probe (#497811-C2, and DapB probe, Advanced Cell Diagnostics, Biotechne®, Hayward, CA) was used to target Insulin 2 mRNA. Sections were counterstained with DAPI and mounted with an antifade mountant (ProLong Gold, Thermo Fisher Scientific), and the images were examined with the confocal microscope.

**Optogenetic-stimulation of the DVC:** Prior to the optogenetic experiments, six 8-week-old male Rip2-ChR2-TdTomato mice were administered intraperitoneally with Temgesic® 0.01 mg/kg (Schering-Plough, NY, USA) 10 min before the start of the surgeries. 4% isoflurane (FORANE, Baxter, Deerfield, USA) to air mixture was used to achieve surgical anesthesia, which was maintained with a 1.0–1.5% to air mixture during the procedure. Optic fiber cannulas, MFC_200/245–0.53_5.5 mm_ZF1.25_C45 (Doric Lenses, Québec, Canada) were implanted unilaterally above the DVC with the following coordinates: −0.0 mm posterior to the occipital suture, 0.25 mm lateral of the midline and −3.4 mm below the surface of the skull, less than 0.5 mm above the target of DVC. Cannulas secured to the skull using dental cement and jewelry anchor screws. After the surgery, animals were single-housed in 1291H cages (425 × 266 × 185 mm, 800cm2) with Sizzle Nest-bedding material (Cat. S1A09 Datesand Group, Bredbury, UK) and maintained in a reversed light/dark cycle with dark onset at 9:00 am. Experimental hardware was purchased from Doric Lenses, Québec, Canada. Fiber optic cannulas were connected to a two-channel led driver (LEDRVP_2CH_1000) with a patchcord (MFP_200/220/900–0.53_1M_FC-ZF1.25(F) together with a bronze 1.25 mm sleeve for mice) via a fiber optic rotary joint (LEDFRJ). The strength of the output 470 nm blue light from the LED-driver was optimized to 10 mW/mm^2^ for each fiber optic cannulae (80–90 mA for mice) using photodiode power sensor (PM100D/S121C Thorlabs Inc., Newton, USA). For the stimulation of hindbrain Rip2-Chr2, a 470 nm light was delivered for 10 ms at a tonic frequency of 10Hz using the Doric Hybrid MultiLED Driver control software. Other tonic frequencies where pre-tested (5–30Hz, data not shown), and 10Hz was determined most effective at altering food intake, and applied to all mice for all optogenetic experiments. For food intake measurements, food was removed 90min before optic stimulation to eliminate random consumption before the test. Food was re-introduced 30min after stimulation start, and stimulation continued for 30min after introduction of food. The shaded area in the graphs showing optogenetic results represents the stimulation period for the stimulated mice and for the non-stimulated mice the sham stimulation which involved inserting the optic fiber without turning on the light source. Time point 0 represents presentation of food. Food intake was measured in home cage at 1, 2 and 24h.

**Pharmacological blockade of stimulation:** To determine whether effects of optical stimulation were insulin signaling dependent an additional group of 8-week-old male and female Rip2-ChR2-TdTomato mice (n = 6) was tested. Similarly to the methods and protocol described for the experiment above, under deep anesthesia, the animals were implanted with optogenetic fiber cannulae and a unilateral infusion guide canula C315GS-2-SP 26 GA cut to 3 mm (Plastics one, Roanoke, USA) 0.2 mm was implanted above the lateral ventricle using the following coordinates: 0.3 mm anterior from the bregma suture, 1.0 mm lateral from the midline and 2.3 mm below the surface of the skull [48] and secured to the skull as mentioned above. After the surgeries and similarly to the experiment above, the mice were single-housed and maintained in a reversed light/dark cycle with dark onset at 9:00 am. The insulin receptor antagonist S961 (Phoenix Pharmaceuticals. Inc. Burlingame, CA USA), 2 μg/μL in 0.5 μL of artificial cerebrospinal fluid, aCSF) was infused (0.5uL/min) into the lateral ventricle using a C315FDS-2/SPC injector cut to 3.2 mm, 30 min prior to optic stimulation, using a micro-infusion pump. The dose of the antagonist was selected based on previous studies using ventricular application of the antagonist [49,50]. The mice were acclimatized at least 5 times to the optogenetic stimulation procedure. The restraint and injection procedure were repeated 3 times before food measurement.

***Ins2-siRNA*:** Forty 6-week-old male C57BL/6J mice were single-housed, weight-matched and divided into two treatment groups. In order to KD Ins2 in the DVC, an AAV2 carrying siRNA targeting the Ins2-gene (AAV2-Ins2-siRNA, Ins2-KD Applied Biological Materials ABM Inc., Richmond, Canada cat. iAAV04339702, titer 1.02∗10^12^ GC/mL) or scrambled virus control (Scrambled AAV siRNA control Virus, Cat. iAAV01502, titer 1.62∗10^12^ GC/mL) were injected bilaterally in the DVC region using the following coordinates: −0.0 mm posterior to the occipital suture, 0.4 mm lateral to the midline and −4.2 mm below the surface of the skull [51]. Aliquots were infused at a speed of 0.1 μL/min with the Hamilton Neuros 10 μL syringe with a 33-gauge needle (Hamilton Co. Reno, NV, USA) to a total volume of 0.5uL, and allowed to diffuse into the parenchyma from the target site for 10 min before needle was removed. In order to remove any experimenter bias during mouse handling, the virus aliquots were coded and distributed by a third party with no connection to the experiment prior to injections. Food and body weight were measured weekly in order to minimize handling. Mice were fed standard chow prior to KD and for 5 weeks following KD. After this period, mice were challenged with HFD (catalog #D12492; Research Diets Inc.; 60% kcal from fat) for 15 weeks.

Biological activity of Ins2-siRNA was assessed in isolated mouse beta islets. Mouse islet isolation was done by injecting liberase^TM^TL (Roche) into the pancreas via the bile duct. The inflated pancreas was dissected and digested in a water bath at 37 °C for 11 min. Islets were handpicked under a stereoscope with a pipette. After allowing the islets to recover in complete medium (RPMI medium supplemented with 10% FBS, 1% penicillin/streptomycin and 10 mmol/L glucose) for 2h. KD of Ins2 was carried out by viral transduction by incubation of islets in RPMI containing 2μL/50 μL of Ins2-KD viral solution (ABM Inc., Richmond, Canada, cat. iAAV04339702, titer 1.02∗1012 GC/mL) or scrambled control (ABM Inc., Richmond, Canada, Cat. iAAV01502, titer 1.62∗1012 GC/mL) overnight. RPMI was added to fill the dish the following morning. Glucose induced insulin secretion: Islets were pre-incubated in Kreb's Ringer buffer (KRB) composed of (mmol/L) NaCL 140, KCL 4.7, CaCl2 2.5, KH2PO4 1.1, MgSO4 1.2, NaHCO3 25, HEPES 10 (pH 7.4 with NaOH) containing 6 mmol/L glucose + 0.1% BSA for 45 min at 37 °C. Islets were then divided in groups – size matched – of 10 islets in tests tubes containing 300 μL KRB plus the desired glucose concentrations and incubated for 1h at 37 °C. Supernatant was collected from each tube into a PCR tube containing 10 μL of aprotinin. For total hormone content measurement, the remaining islets in the test tubes were sonicated for 10 s for total lyse of the cells. Insulin was measured from the supernatant and the lysed islets by ELISA (Mercodia, Uppsala, Sweden).

**Intraperitoneal glucose tolerance test and fasted blood glucose levels and serum insulin:** Given the role of pancreatic insulin, the impact of DVC Ins-KD on glucose tolerance was determined. At 35 days after viral knockdown and at 31 days after initiation of the HFD, mice were fasted for 4h and administered intraperitoneally with glucose (1% (wt/vol), 2 g/kg body weight). Tail vein blood samples (approximately 0.6 μL) were withdrawn by needle incision in the tail vein at 0, 20, 40, 60 and 90 min from mice restrained shortly. Blood glucose concentration was measured using the Bayer Contour® Next XT (Bayer AG, Leverkusen, Germany). Fasted blood glucose levels were measured after a 16h fast and 4h prior to sacrifice. Serum insulin concentration was analyzed using ELISA Mouse insulin kit (N0 10-1247-01; Mercodia, Uppsala, Sweden).

**Thermogenesis:** Core, brown adipose tissue (BAT), and tail temperatures were measured via FLIR T540 thermal camera and analyzed using FLIR tools software as previously described [[46], [52], [53], [54]]. The intrascapular and tail-base region was shaved 4–6h prior to thermography in order to minimize stress during testing but also remove the variable of piloerection. Any experimenter noise was minimized to reduce stress-induced temperature changes. Cage lids were removed and mice were placed on the wire-lid. Three consecutive images were taken swiftly from a distance of one meter (minimum distance) above the animal. The pre-defined smallest circle of the FLIR software was positioned above the scapular region, the tail-base and mid-tail region to obtain the average and the warmest spot in every region of interest (ROI). BAT thermogenic activity was measured from the scapular region, core temperatures in the tail-base/back region and changes in blood perfusion due to cooperative or opposing changes to the core temperatures in the tail [55]. Values obtained from the three consecutive images for each region were averaged. To obtain a stable baseline, measurements were repeated three times over 10 days, and average value is presented.

**Brain tissue and imaging:** RIP-Chr2-YFP or AAV2-Ins2-siRNA injected WT-mice were perfused according to methods described in [56]. Mouse brain samples were frozen slowly on CO_2_-ice after submerging the fixed and sucrose treated brains in O.C.T. solution for 25 min, removing bubbles from the bedding solution prior to the freezing procedure. The block was cut down with approximately a 1 mm layer of bedding material surrounding the tissue. After carefully identifying the DVC region according to [57], 10 μm coronal sections were collected with a Leica 3050S cryostat (Leica Biosystems Nussloch GmbH, Nussloch, Germany) on Superfrost Plus slides (Menzel, Braunschweig, Germany) and mounted using Vectashield H-1200 mounting medium (Vector Labs, Burlingame, CA, USA). Zeiss Axioimager was used to obtain 5-, 10- and 20x epifluorescent images (Carl Zeiss Microscopy GmbH, Jena, Germany).

## 3 Results

### 3.1 Hypothalamic and hindbrain Ins2 gene expression

To determine whether Ins2-promoter cells are present in food intake controlling brain areas, Rip-Chr2-YFP mice were used. In the hypothalamus, abundant expression of cre-induced YFP was localized to the *arcuate nucleus* (Figure 1A, Figure 2, Fig. S3) and the *paraventricular nucleus* (Figure 1B, Fig. S2), but not in the *ventromedial hypothalamus* (Figs. S1 and S2). Interestingly, expression of YFP in the Rip-Chr2-YFP was also present in the nucleus of the solitary tract (NTS) of the dorsal vagal complex (DVC) (Figure 1C, larger image with mouse brain atlas section displayed in Fig. S1). Given the profound impact of DIO on the pancreatic insulin, we hypothesized that brain expression of Ins2, expressed under the Rip-promoter, could also be altered by high-fat DIO (Figure 1D).

After eight weeks of exposure to the HFD, the DIO-mice gained significantly more weight (Figure 1E) and displayed elevated serum insulin levels (Figure 1F) compared to chow-fed control mice (Figure 1E). DIO resulted in reduced Ins2-gene expression in the hypothalamus (Figure 1G), thus in line with our hypothesis. However, Ins2 expression was significantly elevated in the DVC of DIO-mice (Figure 1I). In contrast, preopiomelanocortin (POMC) gene expression, a precursor gene to melanocortin peptides, was not altered by DIO in either the hypothalamus or the DVC (Figure 1H,J), indicating that the changes in Ins2 expression were not a result of global up- or down-regulation of genes in these regions.

### 3.2 Expression of Ins2 mRNA in YFP-labeled cells

RNAscope for *Insulin* 2 mRNA was performed on Rip-Chr2-YFP mouse brain to confirm that cells labeled with YFP driven by Rip promoter express insulin mRNA (Fig. S4). Nearly all NTS cells labeled with YFP had clearly detectable Ins2 mRNA (Fig. S4 C-F). Only 2 out of 148 cells in the randomly selected NTS section were exclusively labeled with YFP (Fig. S4 G) and 2 out of 148 did not seem to show YFP but only the RNA signal (Fig. S4 H). No RNAscope signal was detected in the negative control sample (Fig. S4 L).

### 3.3 Optogenetic stimulation of DVC Ins2-populations increases food intake

In order to determine the impact of the newly identified Ins2-DVC population on feeding behavior, we utilized transgenic mice expressing the light-inducible Chr2 specifically in Rip-populations. To target the DVC, optic fibers were implanted directly above the DVC. Surprisingly, and in contrast to the role hypothesized for pancreatic insulin, optogenetic stimulation of DVC Ins2 neurons induced an orexigenic response. Optogenetic stimulation at 10Hz resulted in increased food intake which peaked at 60min post stimulation onset (Figure 2B–C) (effect: F [1,8] = 6.632, P = 0.03). The DVC Ins2 cell opto-activation did not alter longer-term appetite, as food intake was similar in the stimulated and non-stimulated condition at 24h after reintroduction to food (Supplementary Fig. S5). The location of the optogenetic cannula was confirmed by slicing flash frozen brains coronally through the hindbrain and identifying the cannula tract (representative image shown in Figure 2A, cannula tract indicated by white arrows), which is placed just above the NTS in order for the light cone to reach most of the NTS. Given the placement of the DMX just below the NTS, and the fact that it was also found to have YFP-positive cells it is possible that our stimulation includes the activation of this nucleus as well, albeit given that the cannula is guided just above the NTS, less light is likely to penetrate through the NTS and into the DMX. While the light may also reach the ventral parts of the area postrema (AP), given that we did not see any YFP-positive cells there, it is unlikely to contribute to the behavioral response following optogenetic stimulation. Thus while DMX, as well as other neighboring areas showing YFP label, have the potential to be responsive to the optogenetic activation, it is very unlikely that areas lateral to DVC or below it would be reached by the light cone considering that some studies indicate that the light intensity drops to nearly undetectable levels after traveling through 200 μm of brain tissue [58]. The NTS is 200 μm at the level of our optogenetic fiber placement, thus the stimulation is likely to penetrate through the NTS ventrally but is already too restricted to cover the entire NTS laterally. One recent not yet peer-reviewed study indicates that the light penetration is better than the earlier studies suggest with 10-fold drop in illumination detected at 700 μm [59]. If this is the case, then our stimulation would cover the entire NTS laterally, the DMX, as well as dorsal portions of the hypoglossal nucleus.

**Figure 2:**
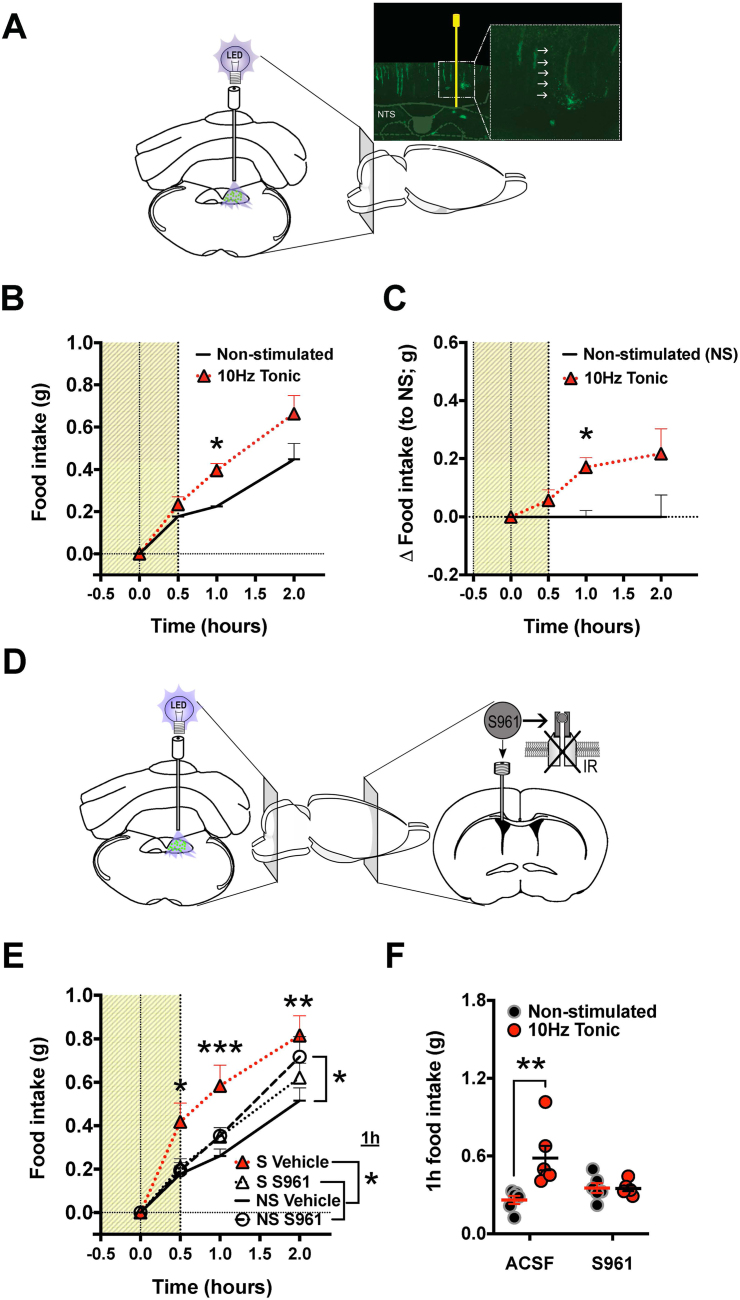
**Hyperphagic effect of optogenetic activation of Ins2 cells in the dorsal vagal complex (DVC) of Rip-Chr2-TdTomato mice** A: Schematic of optogenetic stimulation target and representative confocal image with cannula tract, cannula tract indicated by white arrows in the enlarged square. B: The 10Hz 10 ms tonic stimulation of the DVC increased cumulative food intake with peak consumption at 60min from stimulation onset. C: Results presented as change in food intake from non-stimulated control. In a separate cohort, animals were additionally cannulated to allow infusion of the insulin receptor antagonist, S961. Optogenetic stimulation in the vehicle infused group increased food intake similarly to the first experiment. D–F: However, infusion of the S961 InsR antagonist into the lateral ventricle blocked the effects of the stimulation. E: Main effect of stimulation compared between the non-stimulated and stimulated vehicle condition. At 1h after reintroduction of food, the stimulated vehicle condition group significantly increased intake of food compared to both stimulated and non-stimulated mice infused with S961. At 2h, S961 infusion alone increased food intake compared to non-stimulated vehicle infused control. The shaded area in the graphs represents the stimulation period for the stimulated mice and for the non-stimulated mice the sham stimulation which involved inserting the optic fiber without turning on the light source. Time point 0 represents presentation of food. Artificial cerebrospinal fluid (ACSF), n = 6, ∗p < 0.05, ∗∗p < 0.01, ∗∗∗p < 0.001.

Direct delivery of insulin to the brain has been previously been shown to reduce food intake [24,60] and optical stimulation likely results in release of many different transmitters and peptides produced by this neuronal population. Therefore, we aimed to both replicate the initial unexpected results in a new group of mice, and also to determine whether insulin is implicated in the orexigenic effect. Thus, the dependence of the feeding effect on brain insulin signaling was tested in a new mouse cohort, implanted with both light fibers and ventricular infusion cannulas (Figure 2D). Infusion of the insulin receptor antagonist S961 blocked the effect of optogenetic stimulation on feeding (Figure 2E–F). As in the first experiment, optogenetic activation of Ins2 neurons in the DVC resulted in potent hyperphagia throughout the measured 2h period in the mice injected with vehicle. Mice receiving insulin receptor antagonist did not respond to the optogenetic activation for the first 90min after food reintroduction and hyperphagia returned at the 2h time point if the mice receiving the S961 were not stimulated (Figure 2E) (F [3,20] = 5.1, P > 0.01. Comparing 1h food intake showcases hyperphagic effect present only in vehicle-infused mice, but not in mice infused with S961 (Figure 2F) (F [3,20] = 6.3, P = 0.03). Moreover, the insulin receptor inhibitor itself increased the total food intake at 2h under non-stimulated condition (Figure 2F) (P = 0.03). The effect of both stimulation and S961 was absent at 24h (Supplementary Fig. S5).

### 3.4 Adeno associated virus (AAV)-induced knockdown of DVC Ins2 alters energy balance

The observed Ins2 gene expression response to DIO and the orexigenic response of optogenetic Ins2 neuron activation indicate a divergent effect of the DVC Ins2-gene as compared to literature on pancreatic and hypothalamic insulin/Ins2-neurons. In line with these new findings, we hypothesized that reduction of DVC Ins2 leads to lower body weight via reduced food intake, especially during the DIO challenge. By utilizing Ins2-gene silencing AAV (Ins2-KD) or scrambled AAV control, we first assessed the biological activity of the Ins2-KD vector. Since technological limitations do not currently allow for measuring insulin released from neurons *in vivo*, biological activity of the viral construct was confirmed in mouse pancreatic islets. Insulin secretion was stimulated by 20 mmol/L glucose in isolated mouse pancreatic islets treated with Ins2-KD or with the scrambled control. Insulin secretion was potently stimulated in islets treated with the control virus whereas Ins2-KD treated islets released significantly less insulin (Figure 3A).

**Figure 3:**
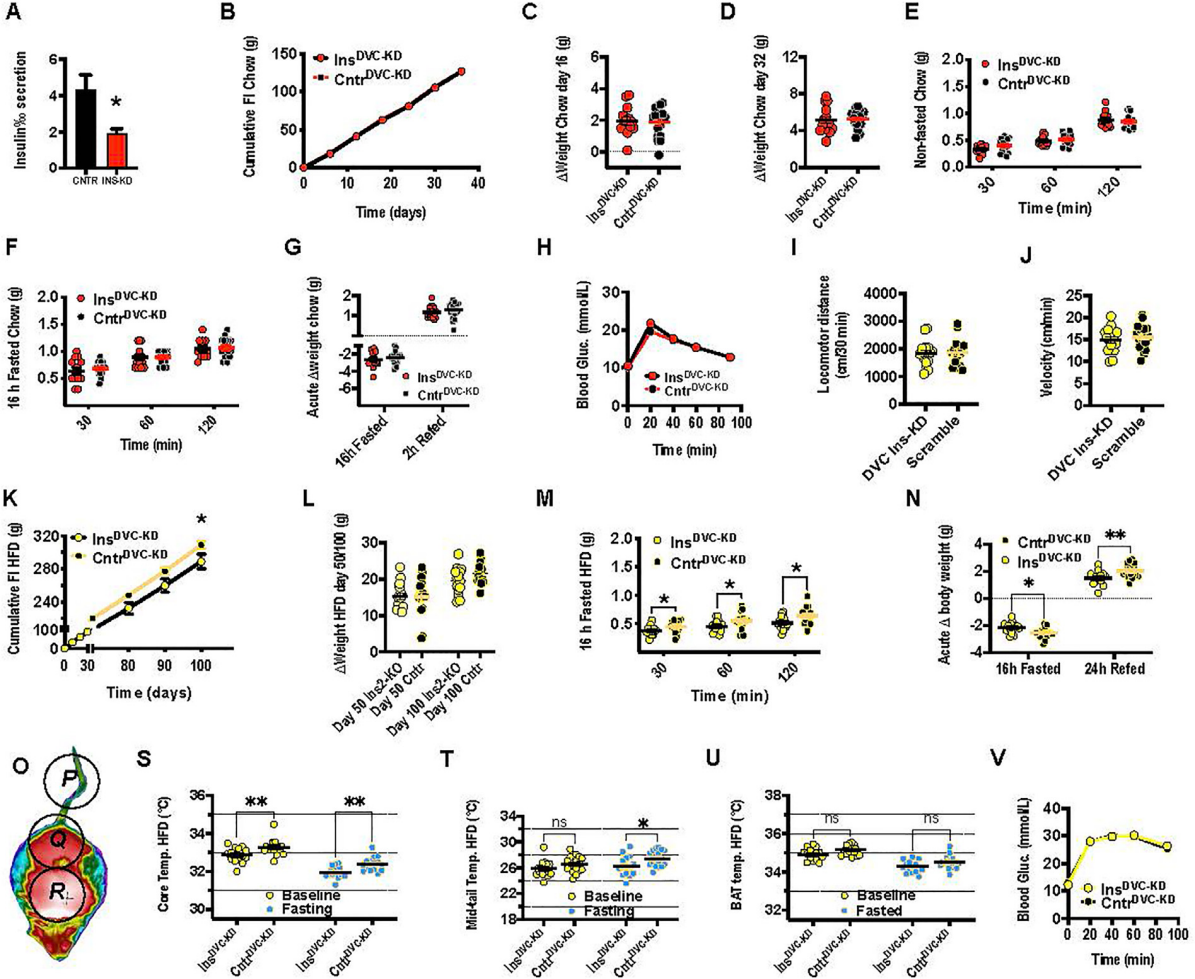
**Ins2-KD in the DVC alters feeding and thermogenesis selectively during high-fat challenge.** A: Isolated mouse pancreatic islets incubated in 20 nmol/L glucose and *in vitro* treated with Ins2-KD virus released significantly less insulin (in % of total insulin content) than scramble treated control, which released insulin at normal expected level. Virally expressed GFP in the NTS is shown in Fig. S6 and reduction in gene expression achieved by the KD in the DMV is shown in Fig. S7. The Ins2-KD had no impact on B: cumulative food intake, body weight gain at C:16 or D: 32 days on the standard chow diet. There was also no effect of the KD in food intake at E: light switch to dark period or F: after a 16 h h fast. Moreover, G: the 16 h fast did not impact weight lost, H: nor did the treatment impact glucose metabolism. There was no difference in I: spontaneous movement or J: velocity while on the HFD. However, K: cumulative food intake on the HFD was significantly decreased at 100 days. There was no significant difference in the L: weight gain at 50 or 100 days, although there was a tendency towards reduced weight gain in the Ins2-KD group (p = 0.1). Similarly, M: food intake after fasting was reduced in the Ins2-KD group at 100 days on HFD. N: The Ins2-KD group lost less weight than the control during the 16 h fast at 100 days on HFD. The difference in weight loss led us to analyze the surface heat in the tail, the back and between the scapula using a FLIR™ camera at 90–100 days on HFD (O). S: Temperatures on the lower back (core, Q) were lower in the Ins2-KD group at baseline and after fasting. T: tail temperatures (mid-tail, P) were lower only after fasting. U: Intrascapular temperatures representing heat production in intrascapular brown adipose tissue (BAT, R), were similar at baseline and after fasting. V: There were no differences between the groups in glucose homeostasis based on the intraperitoneal glucose tolerance test (ipGTT). n = 15–16, ∗p < 0.05, ∗∗p < 0.01 two-way anova.

Next, we targeted the KD to the DVC, in order to evaluate the metabolic and feeding effects of insulin loss in the DVC under normal conditions and under DIO challenge (Figure 3B–V). Virogenetic knockdown of Ins2 in the DVC had no effect on cumulative chow intake, body weight measured at 16 and 32 days post-KD, chow intake during dark cycle, or after a 16h fast (Figure 3B–G). Moreover, the KD did not interfere with fasting-induced body weight change (Figure 3G). The Ins2 DVC KD mice also presented with normal glucose tolerance (Figure 3H). Given that DIO increased levels of Ins2 in the DVC, it is plausible that hindbrain Ins2 plays a more important role under metabolic strain, for example that induced by DIO. In line with this idea, the DIO-induced increase in DVC Ins2-gene expression that we have seen in the wild-type mice (data shown in Figure 1) was likely attenuated in the Ins2-KD group. Thus, while displaying similar locomotor behavior (Figure 3I,J), these mice consumed less HFD (Figure 3K) (interaction: F_(10, 290)_ = 3.6, p = 0.001, KD effect: F [1,29] = 3.6, p = 0.07). Surprisingly, the DVC Ins2-KD did not result in any significant changes in body weight, measured at 50 and 100 days after viral infusion (Figure 3L), although at the 100 days there was a trend to reduced body weight (p = 0.1). The reduced food intake in the KD was not overcome by a 16h fast, as the KD mice consistently ate less food at all refeeding time points (Figure 3M) (KD effect; F [1,29] = 6.1, p = 0.02).

Given the combination of reduced feeding but more stability in body weight as a result of fasting and after refeeding in the KD mice (Figure 3N) (effect; F [1,30] = 11, p = 0.002), we hypothesized that the KD may be engaging compensatory reductions in energy expenditure. Since we did not see any changes in locomotion, we tested whether the mice were compensating for the decreased appetite by reduced energy expenditure via reduced thermogenesis. The KD mice had reduced core body temperature compared to control at baseline and after fasting (Figure 3S) (effect; F [1,30] = 19, p = 0.0002). Tail temperatures in the Ins2-KD group were significantly lower under fasting conditions, potentially indicating heat conservation by reduced blood flow to extremities (Figure 3T) (effect; F [1,30] = 6.2, p = 0.02). Intrascapular BAT temperatures were similar under fasted or fed condition (Figure 3U), indicating that compensatory reduced thermogenesis is not driven by reduced BAT activity. The obese DVC Ins2 KD mice did not show any changes in glucose tolerance, similarly to lean controls (Figure 3V). The AAV2-INS2-siRNA was found throughout the NTS and DMX based on the GFP expression detected in these brain areas (Supplementary Fig. S6). The virus did not spread laterally beyond the NTS. Ventrally from injection site, most of the virus was contained to the NTS, but sparse GFP label was also identified in dorsal portions of the hypoglossal nucleus. As expected at the volume administered (0.5uL) and slow speed of infusion, no signal was detected in the ventral hindbrain. The virus also tended to reduce the expression of Ins2 in the DVC (Supplementary Fig. S7) as expected.

## 4 Discussion

The hypothalamus is well established for its role in energy homeostasis, as well as a site of InsR expression, activation of which changes feeding, body weight, thermogenesis and hepatic glucose production. Hindbrain, just like the hypothalamus, is also privy to blood-borne signals due to its proximity to the leaky BBB, but unlike the hypothalamus it also receives direct input from the vagal gastrointestinal tract signals. It controls feeding behavior and energy expenditure, independently of the hypothalamus or any other forebrain area [[61], [62], [63], [64]]. Insulin receptors have been identified in the NTS and neighboring dorsal motor nucleus of the vagus and in a number of other hindbrain sites [65]. However, insulin-producing neurons in the NTS are entirely unexplored. Current results suggest they can be found throughout the NTS, and more broadly in the DVC, and that they are necessary and sufficient for food intake control.

Insulin of pancreatic and hypothalamic origin has been predominantly shown to reduce food intake [[24], [25], [26],^28^]. This is in line with the fact that pancreatic insulin is released during feeding, to signal high circulating glucose levels [66]. In contrast, infusion of insulin into the brain ventricles also promotes lipogenesis and peripheral fat accumulation [23,24]. Direct activation of DVC Ins2 resulted in an orexigenic response, suggesting that this is a functionally distinct population of Ins2 cells. Given that the optogenetic stimulation likely results in release of different neurotransmitters and neuropeptides harbored by the stimulated cell population, it was reasonable to suspect that the orexigenic effect was driven by a substance other than insulin. However, the orexigenic effect of Ins2 cell activation was blocked by brain-infused InsR antagonist, indicating that the orexigenic effect depends on brain InsR. Together these results suggest the existence of an insulin-InsR brain circuit that is orexigenic in function. The antagonist we used, S961, is highly selective for the InsR, however it can also bind to the IGF1-R. While we used a dose of the antagonist on the lower end of the doses used icv in the literature 1 μg vs 0.5–4.6 μg, it is still possible that it also antagonized some IGF-R. While insulin is thought to be anorexic at least when acting on the hypothalamus [24,27], the feeding effect of IGFs seems less consistent [67]. For example while some studies suggest an anorexic action, the central inhibition of IGF1-R signaling reduces short-term food intake [68]. Thus, we cannot exclude that the hindbrain insulin acts on an orexigenic IGF-R population in addition to or instead of InsR. However, in line with the literature on pancreatic insulin and the CNS InsR knockout, at later time points InsR blockade resulted in an orexigenic effect as well. This could be interpreted as the majority of brain InsR engage an anorexic response. The location of the InsR targeted by the DVC Ins2 cells and what follows, whether they are targeted locally within the DVC or at distal sites, remains to be investigated. However, considering that one previous study reported that injections of insulin into the DVC led to reduced food intake in chow-fed (but not high-fat fed) male rats, receptors outside of the DVC are likely to be targeted by the DVC Ins cells. Moreover, the physiological inputs to this new population of insulin-producing cells, i.e. whether these cells are activated by or Ins2 is elevated by orexigenic signals like neuropeptide Y and ghrelin, or reduced by anorexic signals known to act in the DVC, like glucagon-like peptide-1, is a topic ripe for future investigation.

The orexigenic nature of Ins2 cells in the DVC is further supported by lower food intake in mice with virogenetic Ins2-KD in the DVC. However, this effect was only identified under high-fat diet challenge conditions, suggesting that insulin in the Ins2 DVC cells is dispensable in lean mice. This is not entirely surprising, given that lean mice also showed rather low Ins2 gene expression in the DVC; yet these levels were drastically increased after 2 months of high-fat diet feeding. While high-fat fed DVC Ins2-KD mice reduced their food intake, their body weight only tended to be lower than that of control mice. This was potentially due to compensatory decrease in energy expenditure, possibly stemming from reduced thermogenesis. Interestingly, the reduced thermogenesis did not originate from lower BAT temperature, but rather a reduction in core temperature of the KD mice that was not coupled to a measurable change in BAT temperature. Although locomotory behavior did not differ between the treatment groups, the measurement was conducted in a novel environment, thus we cannot exclude the possibility that locomotory behavior could be altered if measured in a home-cage environment in the KD group.

Pancreatic insulin production is elevated in type 2 diabetes and obesity [35]. However, the Zucker rat, a genetic model of obesity, displays abnormally low insulin levels in the brain, despite the expected hyperinsulinemia in the plasma [21]. This disconnect is also found in patients with Alzheimer's disease, who tend to have elevated plasma insulin but reduced CSF insulin levels [[7], [8], [9]]. Whether this is the result of a reduction in CNS-produced insulin, a defect in insulin transport across the BBB or an entirely different mechanism like volume transmission from epithelial cells in the choroid plexus (EChP's) [69], remains to be tested. To date the literature offers little insight into brain insulin production in obesity, but our data suggests that DIO profoundly alters brain insulin gene expression.

Given the pivotal role of insulin in energy homeostasis regulation, the focus on brain-produced insulin has been predominantly centered around the hypothalamus, but new locations for brain sources of insulin are continuously discovered. Recently Mazucanti and colleagues [69], found that insulin is released from the EChP's. Interestingly, this release was not mediated by glucose, but via the activation of 5HT_2C_-receptors, stimulated by serotonin with origins in the dorsal raphe. Thus, akin to the current data, the EChP's insulin represents a new brain insulin source engaged by factors other than those known to engage pancreatic insulin production and release. Isolated and cultured EChP show both immunoreactivity to insulin and C-reactive peptide. Hence, much like Rip-neurons in the hypothalamus and in the DVC, the insulin promoter is also active in the EChP's [2,70].

The appetite reducing and glucose regulating effects of insulin on the brain are clinically relevant. Brain-directed insulin application (e.g. via intranasal infusions) can reduce food intake and blood glucose [[71], [72], [73]] as well as increase thermogenesis in humans [74]. Intranasal infusions increase insulin levels in the CSF. Human studies indicate that intranasal insulin enhances cognitive performance irrespective of concurrent diabetes [75,76]. The role of insulin within the CNS is evolutionarily conserved, and similar in invertebrates, rodents, and humans. CNS-produced insulin is altered in a pathophysiological context in humans; Alzheimer's disease patients have a dramatically reduced content of brain insulin [77]. Importantly, pancreatic insulin transported across the BBB may not necessarily reach all CNS nuclei expressing InsR [78], whereas intranasal insulin may be transported into most of the brain parenchyma. Consequently, intranasal insulin is likely reaching sites accessible only to endogenously brain-produced insulin. Thus, we must understand the function of this endogenous insulin in order to fully predict the consequences and utility of intranasal insulin or any other brain-reaching insulin administration method as a therapeutic. Understanding the mechanism by which insulin is produced and acts in the brain may aid in the development and refinement of insulin as a therapeutic option to treat obese, diabetic, and even possibly Alzheimer's disease patients.

## Figures and Tables

**figs1:**
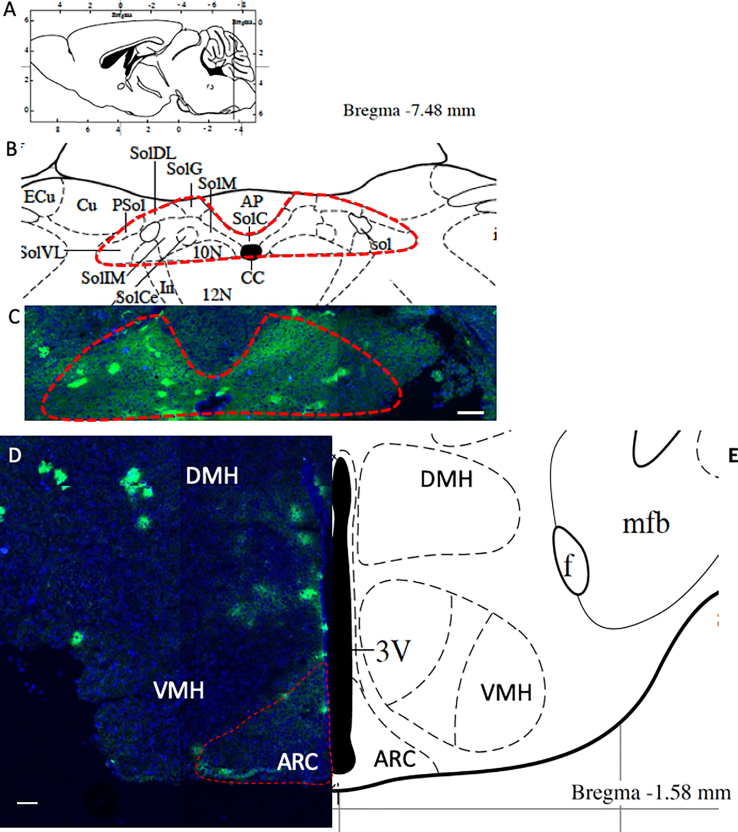


**figs2:**
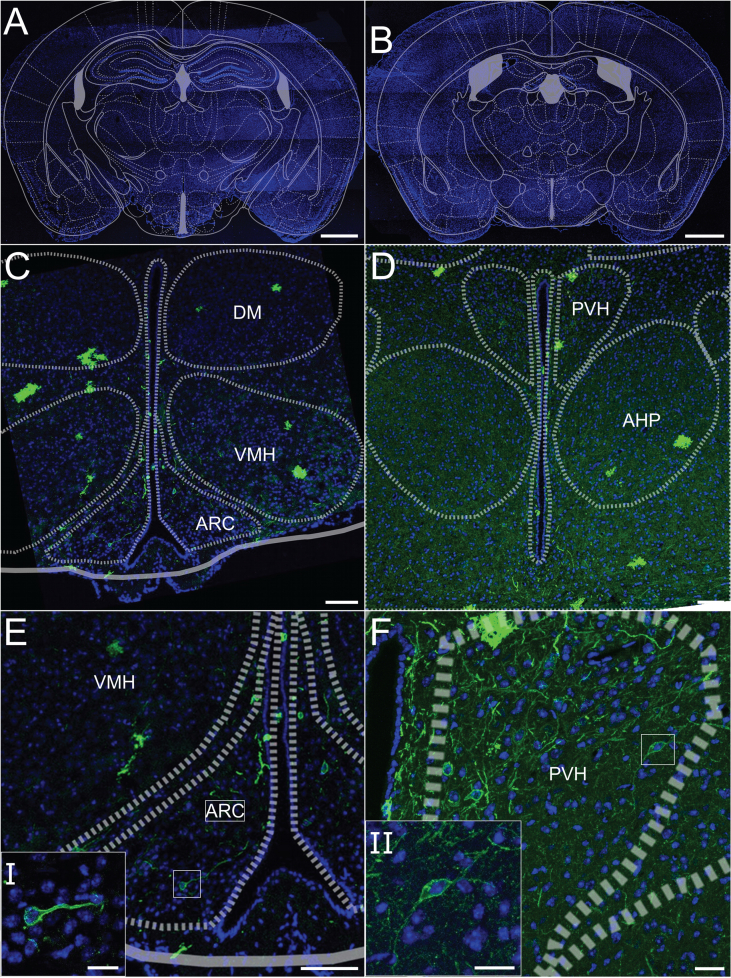


**figs3:**
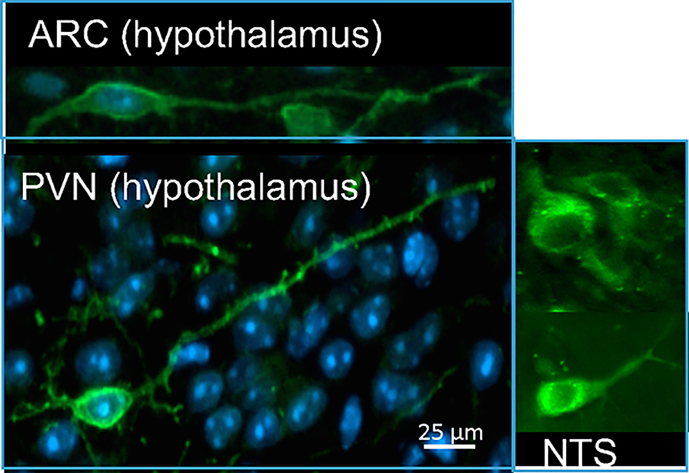


**figs4:**
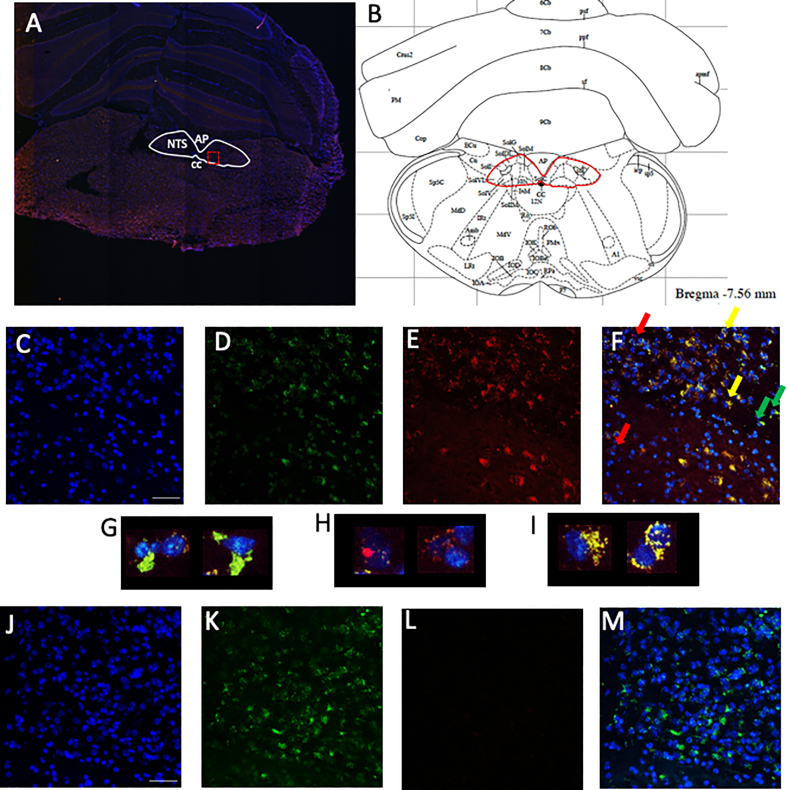


**figs5:**
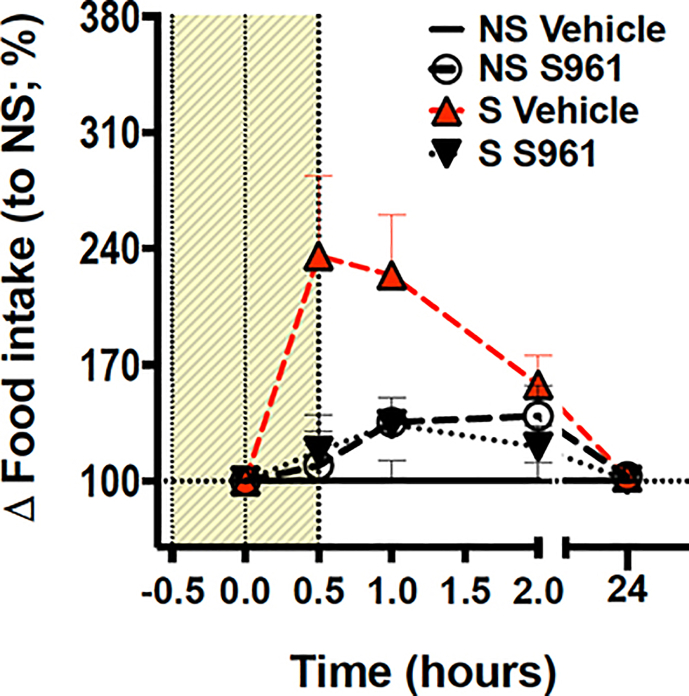


**figs6:**
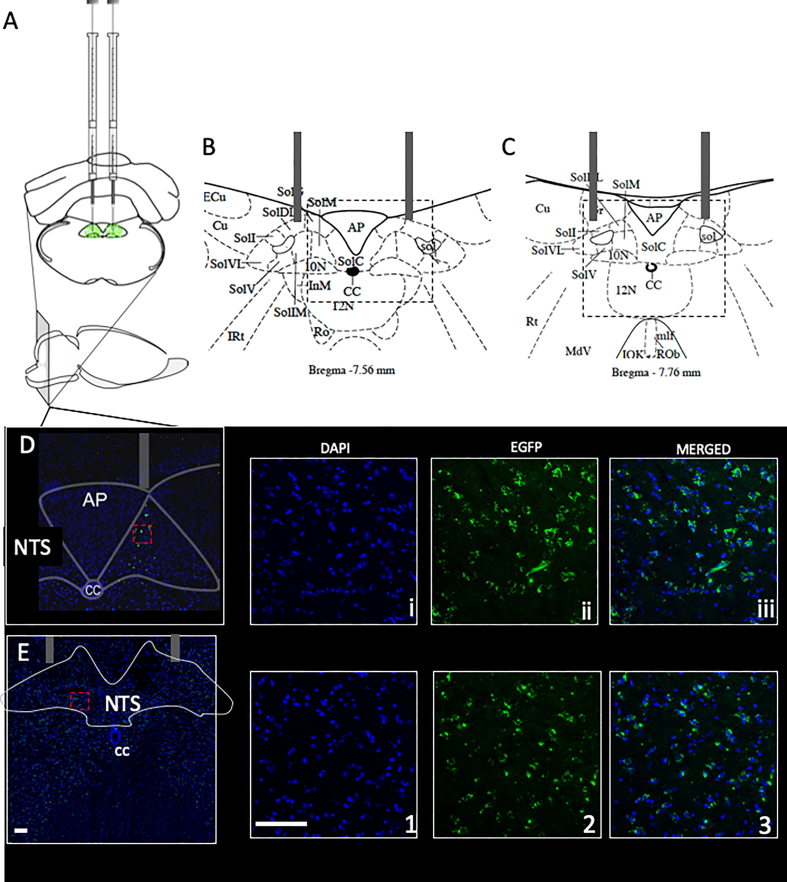


**figs7:**
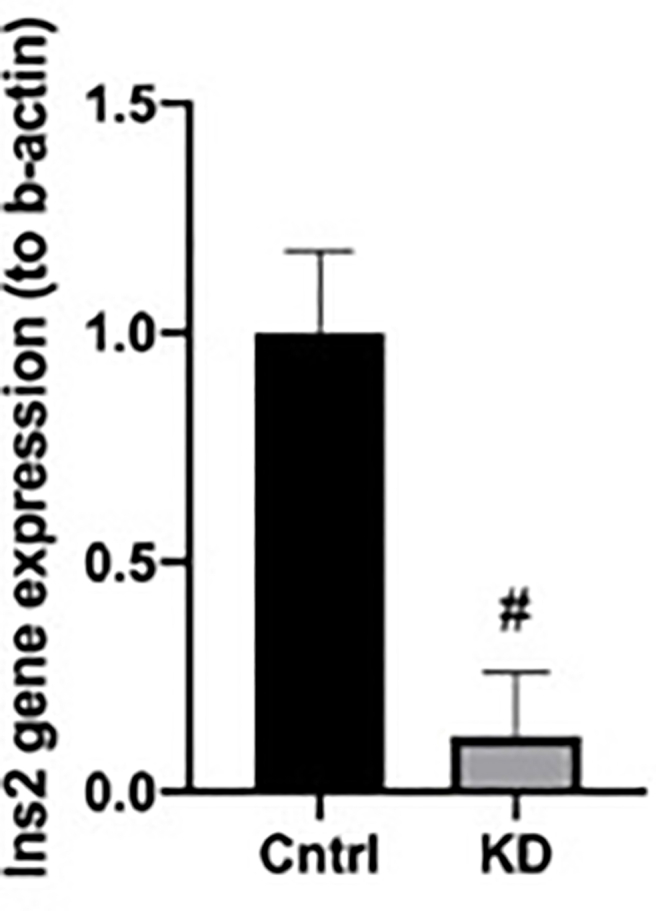


## Data Availability

Data will be made available on request.
